# The Effect of a Freely Available Flipped Classroom Course on Health Care Worker Patient Safety Culture: A Prospective Controlled Study

**DOI:** 10.2196/jmir.5378

**Published:** 2016-07-05

**Authors:** Lowell Ling, Charles David Gomersall, Winnie Samy, Gavin Matthew Joynt, Czarina CH Leung, Wai-Tat Wong, Anna Lee

**Affiliations:** ^1^ Prince of Wales Hospital Department of Anaesthesia and Intensive Care Shatin China (Hong Kong); ^2^ The Chinese University of Hong Kong Department of Anaesthesia and Intensive Care Shatin China (Hong Kong)

**Keywords:** patient safety, critical care, education, professional, education, distance, safety culture

## Abstract

**Background:**

Patient safety culture is an integral aspect of good standard of care. A good patient safety culture is believed to be a prerequisite for safe medical care. However, there is little evidence on whether general education can enhance patient safety culture.

**Objective:**

Our aim was to assess the impact of a standardized patient safety course on health care worker patient safety culture.

**Methods:**

Health care workers from Intensive Care Units (ICU) at two hospitals (A and B) in Hong Kong were recruited to compare the changes in safety culture before and after a patient safety course. The BASIC Patient Safety course was administered only to staff from Hospital A ICU. Safety culture was assessed in both units at two time points, one before and one after the course, by using the Hospital Survey on Patient Safety Culture questionnaire. Responses were coded according to the Survey User’s Guide, and positive response percentages for each patient safety domain were compared to the 2012 Agency for Healthcare Research and Quality ICU sample of 36,120 respondents.

**Results:**

We distributed 127 questionnaires across the two hospitals with an overall response rate of 74.8% (95 respondents). After the safety course, ICU A significantly improved on teamwork within hospital units (*P*=.008) and hospital management support for patient safety (*P*<.001), but decreased in the frequency of reporting mistakes compared to the initial survey (*P*=.006). Overall, ICU A staff showed significantly greater enhancement in positive responses in five domains than staff from ICU B. Pooled data indicated that patient safety culture was poorer in the two ICUs than the average ICU in the Agency for Healthcare Research and Quality database, both overall and in every individual domain except hospital management support for patient safety and hospital handoffs and transitions.

**Conclusions:**

Our study demonstrates that a structured, reproducible short course on patient safety may be associated with an enhancement in several domains in ICU patient safety culture.

## Introduction

Good medical practice is based on the classic maxim of “primum non nocere,” and yet it is estimated that at least 1 in 10 patients may be harmed by adverse events during their hospital stay [1,2]. The landmark US Institute of Medicine’s report in 1999, *To Err is Human: Building a Safer Health System,* ignited the interest in improving patient safety. Studies into harm suggest that a significant proportion of adverse events is preventable [3]. These include prescription mistakes, handover lapses, surgical errors, diagnostic mishaps, and other errors attributable to the human factor [4-7].

Changing and adopting health care technology has been shown to reduce medical errors and improve patient safety [8]. However, technology itself improves patient safety only to a limited extent, and further error reduction requires human factors and organizational change [9]. Clinical human interventions such as additional pharmacist inspection of electronic prescriptions can further reduce medication errors [6]. Using targeted education to change clinical practice seems to be effective as well. Specific interventions adopted for central venous catheter insertions have been shown to reduce central venous access-related infection and improve patient outcome [10]. However, it has also been shown that although targeted educational interventions could improve clinical staff knowledge, this did not translate to improved outcomes [11]. This highlights multiple challenges in studying the effect of education. First, knowledge itself may be a prerequisite for safety culture. However, attitudes and perception are equally important but more difficult to measure and define. Second, education is often not standardized, as such the findings may not be generalizable. Third, it is difficult to conduct blinded randomized trials with appropriate controls. Studies on patient safety education programs have generally not assessed the effectiveness of these interventions with adequate controls and rigor [12].

Recent reports on the failings of a hospital in the United Kingdom have highlighted issues with a lack of an appropriate patient safety culture [13]. The likely causal relationship between poor culture and poor patient care stresses the importance of improving culture to improve standards of care. Therefore, we decided to conduct a prospective controlled study to assess the impact of a standardized, free license, patient safety course on patient safety culture.

## Methods

### Study Design and Hospitals

The study protocol was approved by the Chinese University of Hong Kong Survey and Behavioural Research Ethics Committee. This was a prospective controlled, before and after, study design that used the Hospital Survey on Patient Safety Culture (HSOPSC) questionnaire instrument to evaluate the impact of the BASIC (Basic Assessment and Support in Intensive Care) Patient Safety Course on safety culture. The course was delivered to doctors, nurses, and health care assistants in the Intensive Care Unit (ICU) of hospital A (ICU A) only. In order to control for temporal changes or changes resulting from policies implemented across the entire public hospital system, the questionnaire was also administered to equivalent staff in the ICU of hospital B, a neighboring hospital (ICU B). The two hospitals are publicly funded, located in the same hospital cluster, and share a common cluster chief executive. Hospital A is a tertiary teaching hospital with 1400 beds and 22 ICU beds. Hospital B is an acute general hospital with 600 inpatient beds and 14 ICU beds. A comparison of clinical data between ICU A and ICU B is shown in Table 1.

**Table 1:** Comparison Statistics of ICU A and B.

	Admissions per year	Severity of illness (APACHE III Acute Physiology Score)	Average ICU length of stay	Risk-adjusted hospital mortality ratio
ICU A	1500	50	4 days	0.80
ICU B	600	55	5 days	0.75

### BASIC Patient Safety Course

The BASIC Patient Safety course is a blended learning course that uses a flipped classroom approach in which didactic teaching is carried out prior to participants attending face-to-face teaching. This allows face-to-face time to be dedicated to interactive sessions involving application of the knowledge already acquired. In our course, preparatory material consists of a short printed course manual and e-learning.

The e-learning material comprises short narrated lectures, typically based around a modified clinical case, formative assessment, an interactive electronic lesson, and a video of an incident involving a serious medication error. The e-lectures were created in PowerPoint and Camtasia and were produced as MP4 files so that they could be played on different platforms including Windows, iOS, and Android. The files were uploaded to a Moodle 2.0 platform that was configured to be accessible both on personal computers and mobile devices. Each lecture is supplemented by a formative assessment, in the form of a multiple choice test, which emphasizes the key points covered in the lecture. The system is configured to allow participants to access the assessment only after watching the corresponding lecture for its entire duration.

The interactive lesson is a more complex form of formative assessment. Each participant’s individual pathway through the lesson is dependent on their answers to questions posed during the lesson. Candidates with a poorer understanding of the material take a longer pathway, receiving greater explanation of basic aspects before moving on to more complex issues, while those with a greater understanding rapidly progress to the more complex material. Teaching is thus adjusted to the participant’s needs. All activity on the e-learning site is automatically logged (with the knowledge of participants). Completion of all e-learning is required before attending the face-to-face teaching. Computers are made available to participants who are unable to access the material on their own devices, but no participants required this facility during the study.

Face-to-face teaching consists of small group teaching involving a simulated emergency (to practice communication and team and leadership skills), practicing Situation Background Assessment Recommendation (SBAR) communication, practicing breaking news of an error, discussion of the video shown on the e-learning site, and reflection on patient safety in one’s own unit. This was followed by a group debriefing session. The face-to-face component of the course lasts 3 hours.

The course was written specifically for health care professionals whose primary function is to provide clinical care. It is not aimed at those with a predominantly managerial role. Participants are expected to gain detailed knowledge of the definitions and scope of patient safety, human factors engineering and why it is important to patient safety; cause and reduction of errors, preventing errors leading to harm, cognition, communication with colleagues and patients and the importance of full disclosure, root cause analysis, quality improvement, teamwork, medication safety, and coping with errors. They are expected to enhance the skills required to be an effective team player, understand and learn from errors, understand and manage clinical risk, engage with patients and caregivers, and communicate with full disclosure after adverse events. The course material is available free of charge, and the course is disseminated on a train-the-trainers basis. Facilitators/instructors for the small group teaching are given detailed written guidance on the content for each discussion/tutorial. One instructor is required for every 6 participants.

Between April to December 2011, 117 participants attended the course, of whom 91 nurses and 8 doctors worked in ICU A. No staff from ICU B attended the course. The course was taught predominantly by senior nursing and medical staff from ICU A.

### Measurements

A convenience sample of doctors, nurses, and health care assistants in the ICU from the two hospitals were asked to complete a Hong Kong Chinese version of the HSOPSC before and after the course was implemented to measure their attitudes towards patient safety. The survey consisted of 44 questions with 5-point Likert response scale of agreement of either strongly disagree to strongly agree, never to always, or failing to excellent. Our version contained an extra question about whether the survey participant would feel safe being treated as a patient in the respective hospital. These questions were grouped into 13 dimensions and the overall positive scores were calculated by categorizing strongly agree/agree, excellent/very good, or always/most of time as positive responses. For questions that were negatively worded, positive responses equated to strongly disagree/disagree or never/rarely. The survey also asked the frequency of event reporting by the participant over the past year.

The pre-course (baseline) survey was carried out immediately before the first time the course was run, and the post course survey within 3 months of completion of the series of courses. We wanted to assess the effects of the course on general staff patient safety culture rather than specifically on the attitudes of course attendees. Therefore, the survey respondents were selected randomly from the staff of ICU A and ICU B. Course feedback was collected from participants using anonymized electronic feedback forms.

### Statistical Analyses

We entered the responses into the AHRQ Hospital Survey on Patient Safety Culture Excel tool (version 1.5). Positive responses were coded according to the Survey User’s Guide. For each hospital, the percentage change was estimated as the follow-up percentage minus the baseline percentage. The 95% confidence interval (95% CI) around the positive response percentage for each AHQR patient safety domain was estimated, and baseline results were compared to the 2012 AHRQ ICU sample of 36,120 respondents to provide a contextual reference for interpretation of the applicability of our findings.

Separate generalized estimating equations (GEE) were used to account for the correlation in participants responding to both pre- and post-workshop questionnaires. Separate difference-in-differences models were constructed for each of the 12 AHRQ patient safety domains using participant-level data [14]. The outcome “positive response” was modelled as a function of ICU (A or B), period (baseline or post-intervention), and an interaction term between ICU and period, adjusted for duration of work in Intensive Care (≤10 years vs >10 years). The coefficient for the interaction term in the GEE model indicated whether ICU A improved more or less than ICU B from baseline to follow up. All statistical analysis was carried out using SPSS version 22.0.

## Results

### Response Rates and Participant Characteristics

The total number of questionnaires distributed across the two hospitals was 127 with an overall response rate of 74.8% (95 respondents). Three respondents answered both pre- and post-workshop questionnaires. The pre- and post-intervention response rates from ICU A were 88% (37/42) and 79% (23/29), respectively. The response rates from ICU B were 63% for both pre- (20/32) and post-intervention (15/24) survey.

Of the 95 participants, 78 were registered ICU nurses, 11 patient care assistants, and 6 physicians. Most respondents (90/95, 95%) had direct contact or interactions with patients. Over half (53/95, 56%) had worked in the hospital system for less than 10 years. There was no difference in the proportion of participants working less than or equal to 10 years in the current work area/unit between hospitals: ICU A 58% (33/57) versus ICU B 74% (28/38), *P=*.12.

### Survey Responses

ICU A had lower positive responses at baseline on 7 of 12 domains when compared to baseline responses of ICU B (see Table 2). These included overall perception of safety (*P=*.007), organization learning/continuous improvement (*P=*.03), teamwork within hospital units (*P=*.003), communication openness (*P=*.03), feedback and communication about error (*P<*.001), staffing (*P<*.001), and hospital management support for patient safety (*P=*.02).

After the safety course, ICU A had significantly improved responses in teamwork within hospital units (*P=*.008) and hospital management support for patient safety (*P*<.001), but decreased in the frequency of reporting mistakes (*P=*.006) compared to baseline. For ICU B, there was a decrease in the proportion of positive responses in 6 measured domains during the same period (see Table 2).

There was a significant interaction between ICUs and period, after adjusting for the duration of work in current area/unit, indicating that ICU A showed greater improvement in positive responses than ICU B in 5 domains (see Table 3).

**Table 2:** Unadjusted difference in positive responses at baseline and follow-up between hospitals.

Domain	Baseline responses (%)		Follow-up responses (%)		Changes from baseline (%)^a^			
	ICU B	ICU A	ICU B	ICU A	ICU B	*P*	ICU A	*P*
Frequency of reporting	32/60 (53.3)	43/108 (39.8)	14/43 (32.6)	13/66 (19.7)	-20.8	.04	-20.1	.006
Overall perception of safety	39/80 (48.8)	42/148 (28.3)	21/60 (35.0)	37/92 (40.2)	-13.8	.10	11.9	.06
Supervisor/manager expectations and actions promoting safety	55/80 (68.8)	82/148 (55.4)	33/60 (55.0)	60/92 (65.2)	-13.8	.10	9.8	.13
Organization learning/continuous improvement	45/60 (75.0)	61/111 (55.0)	27/45 (60.0)	44/69 (63.8)	-15.0	.10	8.8	.24
Teamwork within hospital units	71/80 (88.8)	91/147 (61.9)	40/60 (66.7)	72/92 (78.3)	-22.1	.001	16.4	.008
Communication openness	31/60 (51.7)	34/111 (30.6)	12/45 (26.7)	21/69 (30.4)	-25.0	.01	-0.2	.98
Feedback and communication about error	47/60 (78.3)	40/111 (36.0)	18/45 (40.0)	34/69 (49.3)	-38.3	<.001	13.3	.08
Nonpunitive response	19/60 (31.7)	19/111 (17.1)	6/45 (13.3)	10/69 (14.5)	-18.4	.03	-2.6	.64
Staffing	48/80 (60.0)	42/147 (28.6)	24/60 (40.0)	32/90 (35.6)	-20.0	.02	7.0	.26
Hospital management support for patient safety	39/60 (65.0)	44/108 (40.7)	27/45 (60.0)	48/69 (69.6)	-5.0	.60	28.9	<.001
Teamwork across hospital units	41/80 (51.3)	68/144 (47.2)	23/60 (38.3)	39/92 (42.4)	-13.0	.13	-4.8	.47
Hospital handoffs and transitions	40/79 (50.6)	70/143 (49.0)	34/59 (57.6)	43/92 (46.7)	-7.0	.42	-2.3	.74

^a^Follow-up percentage minus the baseline percentage. Denominators for each item are the product of the number of questions in that domain and the number of respondents. Numerators are the total number of positive responses to all questions in that domain.

**Table 3:** Relative risk (95% CI) of improvement in patient safety domains: Baseline to follow-up in hospitals with and without educational intervention.

Domain	Relative risk (95% CI for difference between groups)^a^	*P* value
Frequency of reporting	0.90 (0.33-2.49)	.84
Overall perception of safety	1.94 (1.11-3.37)	.02
Supervisor/manager expectations and actions promoting safety	1.48 (0.99-2.20)	.06
Organization learning/continuous improvement	1.45 (0.96-2.20)	.08
Teamwork within hospital units	1.55 (1.10-2.19)	.01
Communication openness	1.66 (0.73-3.76)	.23
Feedback and communication about error	2.47 (1.28-4.80)	.007
Nonpunitive response	1.68 (0.54-5.18)	.37
Staffing	1.92 (1.15-3.19)	.01
Hospital management support for patient safety	1.88 (1.16-3.04)	.01
Teamwork across hospital units	1.23 (0.75-2.00)	.41
Hospital handoffs and transitions	0.86 (0.44-1.70)	.67

^a^Adjusted for duration of work in current area/unit (≤10 years vs >10 years)

Participants in ICU A were seven times more likely to report “feeling safe being treated in this hospital as a patient” than those in ICU B after adjusting for duration of work in the current area/unit (*P=*.01; see Table 4).

**Table 4:** Response to statement “I would feel safe being treated in this hospital as a patient.”

	Baseline responses (%)		Follow-up responses (%)		Changes from baseline responses (%)		Relative risk^a^ (95% CI)	
	ICU B	ICU A	ICU B	ICU A	ICU B	ICU A		
Feel safe	11/19 (57.9)	14/35 (40.0)	3/15 (20.0)	12/22 (54.5)	-37.9	14.5	7.29 (1.52-34.94)

^a^Interaction effect (risk ratio of improvement from base to follow-up between ICUs, adjusted for duration of work in current area/unit (≤10 years vs >10 years).

Pooled data of all participants from both ICUs indicate that patient safety culture was poorer than the average ICUs in the 2012 AHRQ database (see Table 5). Reponses from both ICUs were lower (at least 5% point difference) for every individual domain except hospital management support for patient safety and hospital handoffs and transitions.

**Table 5:** Domain-level comparative average percentage (95% CI) positive responses of Hong Kong ICUs (N=95) to 2012 AHRQ database (N=36,120).

Domain	Hong Kong ICUs, % (95% CI)	2012 AHRQ ICUs, %
Frequency of reporting	37 (28-47)	59
Overall perception of safety	37 (28-47)	60
Supervisor/manager expectations and actions promoting safety	61 (51-70)	73
Organization learning/continuous improvement	62 (52-71)	72
Teamwork within hospital units	72 (62-80)	84
Communication openness	34 (25-44)	61
Feedback and communication about error	49 (40-59)	60
Nonpunitive response	19 (12-28)	40
Staffing	39 (30-49)	58
Hospital management support for patient safety	56 (46-65)	64
Teamwork across hospital units	45 (36-55)	57
Hospital handoffs and transitions	50 (39-59)	51
Average across domains	47 (38-57)	62

**Figure 1:**
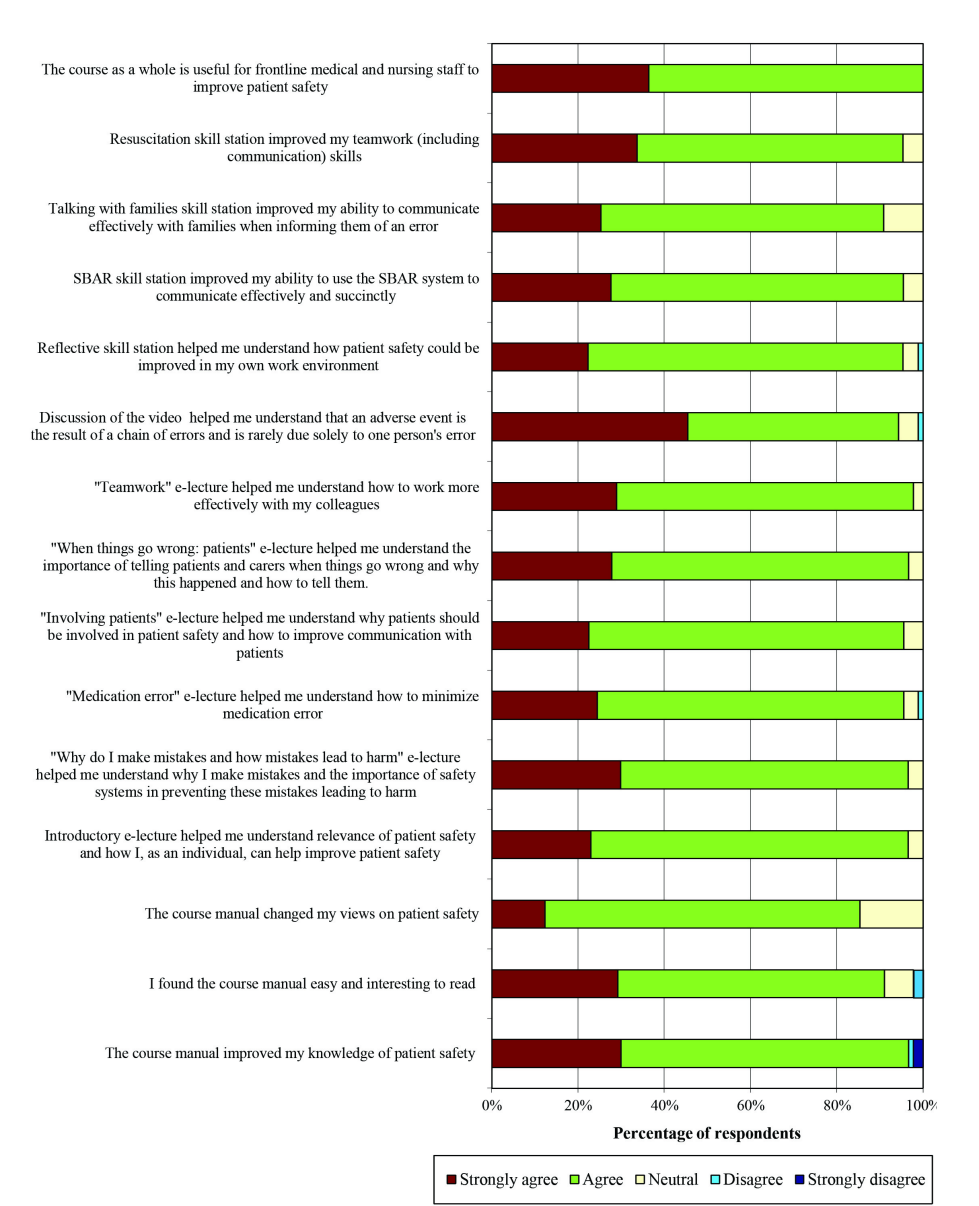
Participants responses to the feedback questionnaire.

Of the 117 safety course participants, 90 (77%) answered the course feedback questionnaire. The vast majority of these 90 participants agreed or strongly agreed with positive statements about the course (see Figure 1), with 32% strongly agreeing and another 56% agreeing that the course was useful to improve patient safety. Notably, participants were as likely to agree or strongly agree with positive statements about the electronic lectures as they were to agree or strongly agree with positive statements about other aspects of the course.

## Discussion

### Principal Findings

Our data suggest that a structured, reproducible, short blended learning course on patient safety may improve perceived ICU patient safety culture. After controlling for duration of working in the respective ICUs, there was a significant improvement in 5 of 12 domains and a trend towards improvement in 2 others in the ICU where the course was given. Furthermore there was a substantial difference in the change of response to the additional statement “I would feel safe being treated in this hospital as a patient” in favor of ICU A.

However, it is notable that there was a deterioration in patient safety culture in ICU B during the study period. This could be due to factors unique to that ICU or hospital or to systemic changes that would also have affected ICU A. If the decline in ICU B was due to systemic factors, then our results suggest that our course not only arrested but largely reversed the deterioration. On the other hand, if the deterioration was due to unique factors affecting only ICU B, then the positive effect of our course could be less than our results would suggest. Nevertheless, if one compares only pre- and post-course results in ICU A in isolation (see Table 2), it can be seen that there were still significant improvements in the domains of teamwork within hospital units and hospital management support for patient safety, and weak evidence supporting an improvement in the feedback and communication about error and overall perception of safety domains.

There was a significant deterioration in the frequency of reporting domain in both ICUs during the study period. In ICU B, this may simply reflect the general deterioration seen across multiple domains. In ICU A, the change is difficult to explain as it is the only domain in which there was a significant reduction. One possibility is that the changes in ICU B did reflect a systemic deterioration in patient safety culture across the two hospitals that was reversed in most, but not all, domains in ICU A by the course. However, we cannot exclude the possibility that this was an inadvertent adverse effect of the course.

### Limitations

Our study has a number of other weaknesses. Similar to all before and after studies, we cannot exclude the possibility of confounding factors that affected only ICU A. Rather than a direct comparison between two ICUs, this study evaluated the temporal change in each unit. Therefore the relevant confounding factors are ones that affect temporal changes rather than the baseline differences between the two ICUs. What we have shown is a temporal relationship between our course and changes in patient safety attitudes, not a causal relationship. The feedback data suggest that the course may have changed attitudes. However, it is possible that the responses of some of the participants from ICU A may have been influenced by the fact that the course was taught by senior staff from the same ICU, even though the feedback was anonymous. Furthermore, any change in attitude may have been due to the involvement of senior staff signaling to other staff that patient safety is an important issue rather than the educational content of the course itself.

We studied only two ICUs; therefore, our results may not be generalizable to other ICUs or to other hospital departments. Previous studies on patient safety culture in Chinese countries such as China and Taiwan showed some important ethnic and cultural factors that may result in differences to western patient safety culture [15-18]. Furthermore, the baseline data suggest that the patient safety culture was poor in both ICUs, relative to ICUs contributing to the AHRQ database, and it is possible that the course may have little effect in units where patient safety culture is well developed. Finally, we have studied only the short-term effect of the course, and although appropriate patient safety culture is considered a pre-requisite to patient safety behavior in practice, this has not been rigorously tested [19].

### Study Strengths

Our study does have the advantage that we studied the effect of a standardized, freely available, educational intervention with a parallel control group [14,20]. The standardized nature of the course both facilitates further research (ie, the same intervention is tested each time) and the applicability of the results (course material may be obtained directly from the authors). Studies of educational interventions that are not in the public domain cannot be reproduced, and it is unclear whether the results can be applied to other individual educational packages. Previous studies of educational interventions to improve patient safety show variable results suggesting that the exact nature of the intervention may be important [10,11,21-24]. In particular, our course incorporates e-learning, interactive modules, and formative assessments. Active involvement and formative assessment are key elements for effective adult learning. A similar educational approach has been used by the Canadian “Managing Obstetric Risks Efficiently” safety program and proven to be effective in advancing safety knowledge (culture was not examined) [25].

Although the course is labor-intensive with a high ratio of instructors to participants, the face-to-face contact time is short as a result of the pre-course reading and e-learning. This facilitates its use as part of in-service training, by minimizing disruption to clinical staffing. It is notable that the e-learning components of the course were highly rated by participants. Although we did not test the specific effect of the e-learning, a study of one of our other courses suggests it enhances learning [26]. The same study revealed that participants value the flexibility of listening to e-lectures at their own convenience and the ability to re-play lectures.

### Conclusions

Our results suggest that the course may improve patient safety culture. Further research is required to establish whether the temporal association can be reproduced when more units are studied in a variety of different cultures and if so, an attempt should be made to determine whether the relationship is causal. In conclusion, introduction of a standardized patient safety course was temporally associated with an improvement in several domains of patient safety culture in a single ICU.

## Abbreviations

AHRQ: Agency for Healthcare Research and Quality
BASIC: Basic Assessment and Support in Intensive Care
GEE: Generalized Estimating Equations
HSOPSC: Hospital Survey on Patient Safety Culture
ICU: Intensive Care Unit
SBAR: Situation Background Assessment Recommendation
